# High Fat Diet-Induced Changes in Mouse Muscle Mitochondrial Phospholipids Do Not Impair Mitochondrial Respiration Despite Insulin Resistance

**DOI:** 10.1371/journal.pone.0027274

**Published:** 2011-11-28

**Authors:** Joris Hoeks, Janneke de Wilde, Martijn F. M. Hulshof, Sjoerd A. A. van den Berg, Gert Schaart, Ko Willems van Dijk, Egbert Smit, Edwin C. M. Mariman

**Affiliations:** 1 Department of Human Biology, NUTRIM School for Nutrition, Toxicology and Metabolism, Maastricht University Medical Center, Maastricht, The Netherlands; 2 Top Institute Food and Nutrition, Nutrigenomics Consortium, Wageningen, The Netherlands; 3 Department of Human Genetics, University Medical Center Leiden, Leiden, The Netherlands; 4 Department of Human Movement Sciences, NUTRIM School for Nutrition, Toxicology and Metabolism, Maastricht University Medical Center, Maastricht, The Netherlands; 5 Department of Internal Medicine, University Medical Center Leiden, Leiden, The Netherlands; Boston University, United States of America

## Abstract

**Background:**

Type 2 diabetes mellitus and muscle insulin resistance have been associated with reduced capacity of skeletal muscle mitochondria, possibly as a result of increased intake of dietary fat. Here, we examined the hypothesis that a prolonged high-fat diet consumption (HFD) increases the saturation of muscle mitochondrial membrane phospholipids causing impaired mitochondrial oxidative capacity and possibly insulin resistance.

**Methodology:**

C57BL/6J mice were fed an 8-week or 20-week low fat diet (10 kcal%; LFD) or HFD (45 kcal%). Skeletal muscle mitochondria were isolated and fatty acid (FA) composition of skeletal muscle mitochondrial phospholipids was analyzed by thin-layer chromatography followed by GC. High-resolution respirometry was used to assess oxidation of pyruvate and fatty acids by mitochondria. Insulin sensitivity was estimated by HOMA-IR.

**Principal Findings:**

At 8 weeks, mono-unsaturated FA (16∶1n7, 18∶1n7 and 18∶1n9) were decreased (−4.0%, p<0.001), whereas saturated FA (16∶0) were increased (+3.2%, p<0.001) in phospholipids of HFD *vs.* LFD mitochondria. Interestingly, 20 weeks of HFD descreased mono-unsaturated FA while n-6 poly-unsaturated FA (18∶2n6, 20∶4n6, 22∶5n6) showed a pronounced increase (+4.0%, p<0.001). Despite increased saturation of muscle mitochondrial phospholipids after the 8-week HFD, mitochondrial oxidation of both pyruvate and fatty acids were similar between LFD and HFD mice. After 20 weeks of HFD, the increase in n-6 poly-unsaturated FA was accompanied by enhanced maximal capacity of the electron transport chain (+49%, p = 0.002) and a tendency for increased ADP-stimulated respiration, but only when fuelled by a lipid-derived substrate. Insulin sensitivity in HFD mice was reduced at both 8 and 20 weeks.

**Conclusions/Interpretation:**

Our findings do not support the concept that prolonged HF feeding leads to increased saturation of skeletal muscle mitochondrial phospholipids resulting in a decrease in mitochondrial fat oxidative capacity and (muscle) insulin resistance.

## Introduction

Insulin resistance and type 2 diabetes are associated with impaired skeletal muscle mitochondrial function [1], [2]. Thus, in skeletal muscle of type 2 diabetic patients both a reduced mitochondrial density and a decreased gene expression of proteins of the mitochondrial respiratory chain have been observed [3], [4], [5], [6]. Interestingly, the reduced skeletal muscle mitochondrial function was already observed in so called pre-diabetic subjects: insulin-resistant offspring of type 2 diabetic subjects, at risk for developing type 2 diabetes in later life [1], [7].

The putative factors involved in impairing mitochondrial function in relation to insulin resistance and type 2 diabetes are not completely understood. However, increased lipid supply to the muscle is considered a potential cause. Indeed, acute elevation of plasma free fatty acids (FA) by lipid infusion in healthy subjects decreased the expression of skeletal muscle *Pgc1α*, *Pgc1*β and other genes involved in mitochondrial metabolism [8], [9]. Lipid infusion in humans also led to a reduced insulin-stimulated increase in ATP synthase flux in skeletal muscle as assessed by NMR spectroscopy [10]. In line with these observations, consumption of a 3-day high-fat diet (HFD) reduced the expression of mitochondrial oxidative genes as well as *Pgc1α* and *Pgc1*β in skeletal muscle of young healthy subjects [11]. Furthermore, it was also shown that prolonged consumption of a of high-fat/high-sucrose diet in mice resulted in a reduction of skeletal muscle mitochondrial capacity [12].

In contrast to these observations, several rodent studies have also shown that a HFD increases, rather than decreases whole-body lipid oxidation, mitochondrial FA oxidation, mitochondrial respiration, activity of mitochondrial enzymes and markers for mitochondrial density. Despite this increase of mitochondrial density and oxidative capacity, the consumption of a HFD did induce insulin resistance [13], [14], [15], [16], [17]. These findings question the concept that mitochondrial dysfunction is a primary cause of insulin resistance [18], [19]. This is also underscored by the study of Bonnard et al. [12], showing mitochondrial dysfunction in skeletal muscle after 16 weeks, but not after 4 weeks high-fat/high-sucrose feeding while muscle insulin resistance was observed after both 4 and 16 weeks of dietary intervention.

Hence, although the primary role of skeletal muscle dysfunction in the pathogenesis of insulin resistance and type 2 diabetes the disease is under debate 20,21,22, it is generally accepted that a mitochondrial defect, possibly secondary to an increase fat intake, does exist in this disease.

The link between an increased intake of dietary fat and changes in mitochondrial functional capacity possibly resides in the FA composition of mitochondrial phospholipids. In this context, it was shown that aging increases the proportion of SFA in rat testis mitochondria, which was accompanied by a decreased activity of the mitochondrial respiratory enzymes [23]. Furthermore, we recently found that a 4-week palm oil-based HFD resulted in an increased saturation of skeletal muscle phospholipids [16]. Although a relation between insulin sensitivity and the FA composition of skeletal muscle membrane phospholipids has been demonstrated [24], [25], [26], [27], it is currently unknown if changes in the FA composition of skeletal muscle mitochondrial phospholipids contribute to the development of mitochondrial dysfunction and insulin resistance.

Therefore, the aim of the present study was to test the hypothesis that a HFD induces an increased saturation of the skeletal muscle mitochondrial phospholipids resulting in impaired mitochondrial respiratory capacity and possibly insulin resistance. To study mid-term and long-term HFD-induced effects, C57BL/6J mice were fed a palm oil-based HFD for 8 or 20 weeks. Specifically, we analyzed the FA composition of skeletal muscle mitochondrial phospholipids, performed high-resolution respirometry to assess oxidation of pyruvate and fatty acids in isolated skeletal muscle mitochondria and measured markers for muscle mitochondrial density and insulin sensitivity.

## Results

### Body mass and net energy intake

Table 1 shows body mass and parameters of energy metabolism of HFD mice and LFD mice at 7 and 19 weeks. Body mass was significantly higher in HFD mice than in LFD mice and was significantly higher at 19 weeks than at 7 weeks. Furthermore, the time-induced increase in body weight was significantly more pronounced in HFD *vs.* LFD animals. These changes in body weight were accompanied by a significantly higher gross energy intake in HFD mice as compared to LFD mice. Additionally, gross energy intake significantly increased with time (7 *vs.* 19 weeks) while neither diet nor time significantly changed faecal energy loss. Consequently, net energy intake was significantly higher in HFD mice as compared to LFD mice. Furthermore, net energy intake significantly increased between 7 weeks and 19 weeks.

**Table 1:** Energy metabolism in week 7 and week 19 of diet intervention.

	Week 7	Week 7	Week 19	Week 19	*P* value	*P* value	*P* value
	LFD	HFD	LFD	HFD	diet	time	diet * time
Body mass (g)	28.1±0.9	32.5±1.5	31.4±0.6	42.9±2.0	<0.001	<0.001	0.014
Food intake (g/week)	19.1±2.0	16.5±1.9	22.0±1.0	19.1±1.8	0.013	0.013	0.900
Gross energy intake (kJ/week)	261±27	388±45	300±14	450±43	<0.001	0.020	0.547
Feces (g/week)	1.8±0.3	1.8±0.2	2.3±0.2	2.2±0.2	0.656	0.005	0.961
Energy loss (kJ/week)	31.0±5.3	32.2±4.9	36.1±6.9	35.6±4.1	0.916	0.150	0.753
Net energy intake (kJ/week)	229±23	356±41	263±20	415±41	<0.001	0.023	0.493

Values are means ± SE (*n* = 8). HFD, high fat diet; LFD low fat diet.

### Fatty acid composition of skeletal muscle mitochondrial phospholipids

#### Changes in the relative amounts of saturated, mono-unsaturated and poly-unsaturated fatty acids

After the dietary interventions, we isolated skeletal muscle mitochondria and determined the phospholipid composition of the mitochondrial membranes.

Table 2 shows the relative amounts of SFA, mono-unsaturated FA (MUFA), PUFA, the n-3 *vs.* n-6 PUFA ratio and the unsaturation index in skeletal muscle mitochondrial phospholipids of HFD mice and LFD mice, respectively. Mitochondrial phospholipids of HFD mice contained significantly more SFA than mitochondrial phospholipids of LFD mice. Additionally, a significant diet * time effect was found, indicating decreases in SFA over time in HFD mice, but not in LFD mice (HFD: 42.8% *vs.* 41.3% and LFD: 39.8% *vs.* 40.2% in 8-week *vs.* 20-week). Relative amounts of MUFA were lower in mitochondrial phospholipids of HFD *vs.* LFD mice. In contrast, relative amounts of PUFA were significantly higher in mitochondrial phospholipids of HFD mice *vs.* LFD mice. In addition, a significant diet * time interaction was found, revealing a time-related increase in PUFA in HFD mice, but not in LFD mice (HFD: 46.1% *vs.* 47.4% and LFD: 45.4% *vs.* 44.4% in 8-week *vs.* 20-week).

**Table 2:** Relative amounts of SFA, MUFA, PUFA, n-3 PUFA, n-6 PUFA, the ratio n-3 *vs.* n-6 and the unsaturation index in mitochondrial phospholipids from hind limb muscles.

	Week 8	Week 8	Week 20	Week 20	*P* value	*P* value	*P* value
	LFD	HFD	LFD	HFD	diet	time	diet * time
SFA	39.8±0.3	42.8±0.3	40.2±0.1	41.3±0.3	<0.001	0.038	0.001
MUFA	15.0±0.1	11.0±0.1	15.4±0.2	11.3±0.1	<0.001	0.010	0.470
PUFA	45.2±0.4	46.1±0.3	44.4±0.2	47.4±0.2	<0.001	0.421	0.002
n-3 PUFA	17.1±0.2	16.0±0.3	17.2±0.4	16.3±0.4	0.004	0.465	0.797
n-6 PUFA	27.6±0.5	29.6±0.2	26.7±0.4	30.6±0.3	<0.001	0.921	0.017
n-3 *vs.* n-6	0.63±0.02	0.54±0.01	0.65±0.02	0.53±0.02	<0.001	0.572	0.343
UI	209±1.3	208±1.9	208±1.3	213±1.9	0.400	0.185	0.093

Values are relative amounts expressed as percentage (moles of a FA as a percentage of total mol of FA in mitochondrial phospholipids) and are means ± SE (*n* = 8). HFD, high fat diet; LFD low fat diet; MUFA, mono-unsaturated fatty acids; PUFA, poly-unsaturated fatty acids; SFA, saturated fatty acids; UI, unsaturation index.

Whereas the relative amount of n-3 PUFA was significantly lower in mitochondrial phospholipids of HFD mice than in mitochondrial phospholipids of LFD mice, the amount of n-6 PUFA was significantly higher in HFD mice *vs.* LFD mice. Furthermore, a significant diet * time interaction for n-6 PUFA was found indicating an increase in n-6 PUFA over time in HFD mice, but not in LFD mice. We also observed a significantly lower n-3 *vs.* n-6 PUFA ratio in HFD mice *vs.* LFD mice. Finally, a tendency for a diet * time interaction for the unsaturation index was found (*p* = 0.09), with a time-related increase in HFD mice *vs.* LFD mice (HFD: 208 *vs.* 213, LFD: 209 *vs.* 208 in 8-week vs. 20-week).

In summary, relatively less MUFA were found in skeletal muscle mitochondrial phospholipids of HFD mice *vs.* LFD mice at both 8 and 20 weeks. This reduction in MUFA was paralleled by HFD-induced increases in both SFA and PUFA. However, the increase in SFA was more pronounced at 8 weeks, whereas the increase in PUFA was more prominent after 20 weeks of dietary intervention. Furthermore, a lower n-3 *vs.* n-6 PUFA ratio was found in HFD mice in comparison with LFD mice while the unsaturation index increased with time in HFD- but not in LFD mice.

#### Changes in the relative amounts of individual fatty acids

Only FA with a relative amount >2% in skeletal muscle mitochondrial phospholipids were further analyzed. The most abundant FA were palmitic acid (16∶0), palmitoleic aid (16∶1n7), stearic acid (18∶0), vaccenic acid (18∶1n7), oleic acid (18∶1n9), linoleic acid (18∶2n6), arachidonic acid (20∶4n6), docosapentaenoic acid (22∶5n6) and docosahexaenoic acid (22∶6n3) as shown in Table 3.

**Table 3:** Relative amount of most abundant fatty acids in mitochondrial phospholipids from hind limb muscles.

	Week 8	Week 8	Week 20	Week 20	*P* value	*P* value	*P* value
	LFD	HFD	LFD	HFD	diet	time	diet * time
16∶0	24.0±0.2	27.2±0.2	24.8±0.2	26.5±0.2	<0.001	0.933	0.002
16∶1n7	3.1±0.1	1.2±0.1	3.2±0.1	1.3±0.1	<0.001	0.141	1.000
18∶0	14.4±0.1	14.7±0.2	14.0±0.2	13.9±0.2	0.648	0.002	0.291
18∶1n7	5.1±0.0	3.6±0.1	4.8±0.1	3.4±0.0	<0.001	0.002	0.447
18∶1n9	6.1±0.1	5.6±0.1	6.7±0.1	6.0±0.1	<0.001	<0.001	0.280
18∶2n6	10.0±0.2	10.2±0.3	9.4±0.3	11.1±0.3	0.001	0.547	0.009
20∶4n6	11.7±0.2	12.4±0.1	11.3±0.2	12.0±0.2	<0.001	0.025	1.000
22∶5n6	3.4±0.1	4.5±0.1	3.6±0.1	4.9±0.2	<0.001	0.032	0.380
22∶6n3	15.6±0.2	14.6±0.2	16.0±0.3	15.0±0.3	0.002	0.206	0.916

Values are relative amounts expressed as percentage (moles of a FA as a percentage of total mol of FA in mitochondrial phospholipids) and are means ± SE (n = 8). FA, fatty acids; HFD, high fat diet; LFD, low fat diet.

Mitochondrial phospholipids of HFD mice contained significantly more 16∶0 than mitochondrial phospholipids of LFD mice. Interestingly, a significant diet * time effect was found for 16∶0 with an increase in 16∶0 over time in LFD mice and a decrease in HFD mice (LFD: 24.0% *vs.* 24.8% and HFD: 27.2% *vs.* 26.5% in 8-weeks *vs.* 20-weeks). For 18∶0, a significant time-related decrease was found. In addition, the relative amounts of 16∶1n7, 18∶1n7 and 18∶1n9 were significantly lower in mitochondrial phospholipids of HFD mice than in LFD mice. Furthermore, the relative amounts of the n-6 PUFA 18∶2n6, 20∶4n6 and 22∶5n6 were all significantly higher in HFD *vs.* LFD mice. For 18∶2n6, a significant diet * interaction effect was found, with an increase in 18∶2n6 with time in HFD mice but not in LFD mice (HFD: 10.2% *vs.* 11.1% and LFD: 10.0 *vs.* 9.4% in 8-week *vs.* 20-week). Finally, the relative amount of 22∶6n3 was significantly lower in mitochondrial phospholipids of HFD mice *vs.* LFD mice.

In summary, skeletal muscle mitochondrial phospholipids of HFD mice contained relatively more 16∶0, 18∶2n6, 20∶4n6 and 22∶5n6 and less 16∶1n7, 18∶1n7, 18∶1n9 and 22∶6n3 in comparison with LFD skeletal muscle mitochondria. Furthermore, the HFD-induced increase in the relative abundance of 16∶0 was more pronounced at week 8 of the dietary intervention whereas the HFD-induced increase in 18∶2n6 was most pronounced at week 20. The absolute amounts of all described FA are shown in Supporting Information S1.

### High-resolution respirometry

Next, we tested whether the changes in the phopholipid composition of skeletal muscle mitochondria affected the mitochondrial oxidation of both a carbohydrate-derived substrate (pyruvate) and a lipid-derived substrate (palmitoyl-CoA + carnitine).

**Figure 1:**
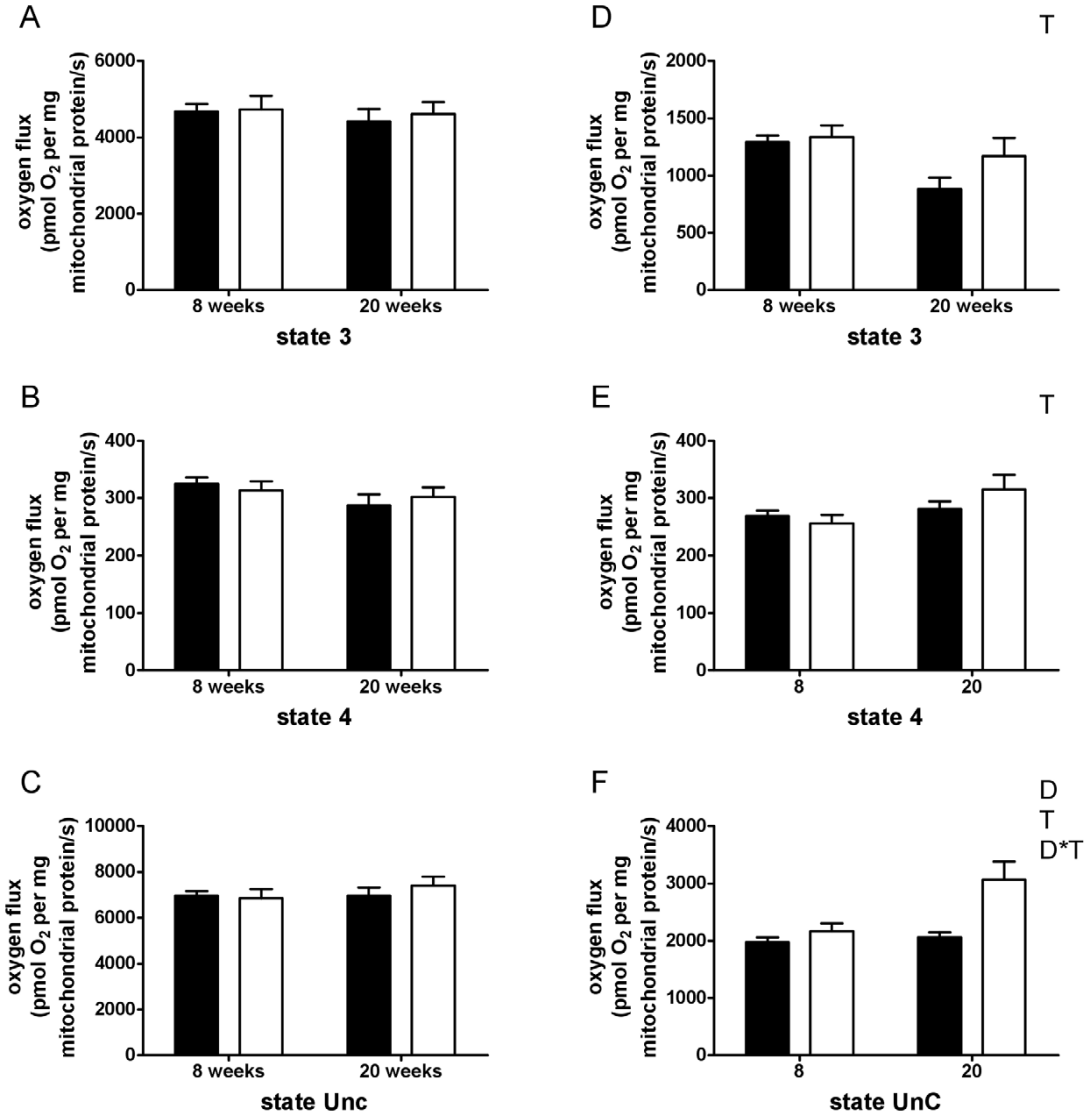
Respiration rates of isolated mitochondria from mouse skeletal muscle on pyruvate and palmitoyl-CoA+carnitine. (A) ADP-stimulated (state 3) respiration on pyruvate, (B) oligomycin-insensitive (state 4) respiration on pyruvate, (C) maximally uncoupled (state UnC) respiration on pyruvate, (D) state 3 respiration on palmitoylCoA + carnitine, (E) state 4 respiration on palmitoyl-CoA + carnitine and (F) State UnC respiration on palmitoyl-CoA + carnitine. Black and white bars represent LFD mice and HFD mice, respectively. Values are means ± SE (n = 7–8). D, significant diet effect with *p*<0.01; T, significant time effect with *p*<0.05 in state 3 and state 4 and with *p*<0.01 in state UnC; D*T, significant diet * time effect with *p*<0.05; HFD, high fat diet; LFD, low fat diet; TA, tibialis anterior; UnC, uncoupled.

Neither diet, nor time significantly changed mitochondrial oxidation of pyruvate, i.e. ADP-stimulated (state 3), oligomycin-insensitive (state 4) and maximally uncoupled respiration (state UnC) were comparable between diets and time points (Figure 1A–C). Consequently, also the respiratory control ratio (RCR), which is defined as state 3 over state 4 respiration, was unaltered upon either time or diet and averaged 14.4±0.5 *vs.* 15.0±0.5 after 8 weeks LFD *vs.* HFD, respectively, while RCR values after 20 weeks of dietary intervention averaged 15.4±0.6 *vs.* 15.3±0.7 in LFD *vs.* HFD, respectively.

State 3 respiration on palmitoylCoA + carnitine was higher in HFD mice than in LFD mice (8-week: +3.8% and 20-week: +33%), although this difference was not statistically significant (*p* = 0.127) (Figure 1D). Additionally, we observed a significant time-induced decrease in state 3 respiration, whereas state 4 respiration significantly increased with time (Figure 1E). These changes resulted in a significant (*p*<0.001) time-induced reduction in RCR (LFD: −33% and HFD: −30%). Diet increased the RCR (8-week: +10% and 20-week: +16%), but this increase did not reach statistical significance (*p* = 0.145).

Maximally uncoupled respiration was significantly higher in HFD *vs.* LFD mice (8-week: +9.5% and 20-week: +49% with *p*<0.01). Also a significant time-induced increase was observed for maximally uncoupled respiration. Finally, a significant diet * time effect was found, indicating a stronger increase in maximally uncoupled respiration in HFD *vs.* LFD mice over time (+42% *vs.* +4.0% in HFD *vs.* LFD) (Figure 1F).

### Parameters of mitochondrial density

Besides assessing the mitochondrial oxidation of a carbohydrate- and a lipid-derived substrate, we also studied the effects of the dietary intervention on the amount of muscle mitochondria. To this purpose, we analyzed several parameters for mitochondrial density in the tibialis anterior (TA) muscle: 1) mitochondrial DNA copy number; 2) protein levels of structural subunits of the five complexes of the respiratory chain (OxPhos proteins) and 3) citrate synthase (CS) and hydroxyacyl-CoA dehydrogenase (HAD) activity (Table 4).

**Table 4:** Parameters for mitochondrial density in the TA muscle.

	8 weeks	8 weeks	20 weeks	20 weeks	*P* value	*P* value	*P* value
	LFD	HFD	LFD	HFD	diet	time	diet * time
Mitochondrial DNA copy number (AU)	0.96±0.07	0.99±0.11	1.36±0.14	1.19±0.05	0.469	0.007	0.336
Sum of the 5 complexes of respiratory chain (AU)	4.6±0.4	6.5±0.5	7.2±0.7	12.8±0.9	<0.001	<0.001	0.012
I	0.82±0.13	1.57±0.28	1.69±0.16	2.39±0.15	0.001	<0.001	0.892
II	0.81±0.14	1.46±0.22	1.09±0.09	2.02±0.22	<0.001	0.028	0.439
III	0.97±0.20	1.59±0.11	1.61±0.12	2.97±0.30	<0.001	<0.001	0.076
IV	0.25±0.04	0.28±0.02	0.55±0.07	1.08±0.12	0.001	<0.001	0.003
V	1.80±0.36	1.64±0.28	2.30±0.56	4.38±0.43	0.033	0.001	0.014
CS (µmol/min/g protein)	29.0±1.8	31.5±2.1	31.1±2.6	32.6±2.7	0.399	0.488	0.828
HAD (µmol/min/g protein)	8.7±1.2	11.3±1.1	10.2±1.3	12.5±1.5	0.064	0.303	0.926

Parameters in the TA muscle in LFD mice and HFD mice at 8 weeks and 20 weeks. Values are means ± SE (n = 6–8). OxPhos protein levels were normalized for Gapdh protein content. CS, citrate synthase; HAD, β-hydroxyacyl-CoA dehydrogenase; HFD, high fat diet; LFD, low fat diet; TA, tibialis anterior.

Surprisingly, we observed only a significantly time-related increase in mitochondrial DNA copy number, while diet remained without effect.

In contrast, Western blotting of the OxPhos proteins (Supporting Information S2) showed significantly higher protein levels in HFD *vs.* LFD mice (8-week: +41% and 20-week: +78% for the total sum of the 5 OxPhos proteins assessed). Furthermore, a significant diet * time effect was observed, with a stronger increase in protein levels over time in HFD *vs.* LFD mice (+97% *vs.* +57% in HFD *vs.* LFD).

We also measured the activity of two mitochondrial enzymes, CS and HAD. However, neither diet, nor time significantly influenced CS activity. HAD activity was higher in HFD mice than in LFD mice (8-week: +30% and 20-week: +23%), although this difference did not reach statistical significance (*p* = 0.064). No correlations were found between the three different parameters.

In addition, we also assessed the protein levels of porin, a mitochondrial housekeeping protein, in the TA muscle (Supporting Information S2). However, protein levels were not significantly affected by any of the dietary interventions and averaged 13.1±2.3 AU (LFD) *vs.* 13.1±1.4 AU (HFD) after 8 weeks and 13.2±1.3 AU (LFD) *vs.* 16.1±2.1 AU (HFD) after 20 weeks of dietary intervention. Finally, expression of the OxPhos proteins relative to porin (table 5) revealed that the increase in OxPhos protein levels upon HFD was not due to an increase “per mitochondrion”, but most likely reflect a change in mitochondrial density.

**Table 5:** OxPhos proteins in TA muscle relative to porin, a mitochondrial housekeeping protein.

	Week 8	Week 8	Week 20	Week 20	*P* value	*P* value	*P* value
	LFD	HFD	LFD	HFD	diet	time	diet * time
I	1.19±0.17	1.35±0.14	1.28±0.26	1.35±0.22	0.598	0.815	0.840
II	0.91±0.05	1.24±0.12	0.89±0.19	1.14±0.17	0.077	0.695	0.807
III	1.73±0.45	1.65±0.28	1.39±0.25	1.59±0.17	0.848	0.496	0.632
IV	1.70±0.41	1.53±0.20	2.42±0.30	3.41±0.49	0.277	0.003	0.137
V	3.57±0.84	1.82±0.37	2.05±0.56	2.63±0.47	0.310	0.531	0.054

Values are means ± SE (*n* = 4–5). HFD, high fat diet; LFD, low fat diet.

### Plasma parameters and glucose homeostasis

Finally, to test whether the effects of the dietary intervention on muscle mitochondrial metabolism were related to changes in insulin sensitivity, we measured plasma glucose and insulin levels and calculated the HOMA-IR index (Table 6). Additionally, we also determined plasma leptin levels.

**Table 6:** Plasma levels of leptin, glucose and insulin after 8 weeks and 20 weeks of diet intervention.

	Week 8	Week 8	Week 20	Week 20	*P* value	*P* value	*P* value
	LFD	HFD	LFD	HFD	diet	time	diet * time
Fasting leptin (ng/ml)	7.3±1.2	57.2±3.4	14.9±2.7	65.8±5.4	<0.001	0.031	0.902
Fasting glucose (mmol/l)	8.2±0.4	10.3±0.3	8.2±0.3	9.2±0.3	<0.001	0.120	0.110
Fasting insulin (µU/ml)	12.3±2.5	32.6±3.9	17.6±5.2	88.8±9.9	<0.001	<0.001	<0.001
HOMA-IR	4.4±0.7	15.2±2.0	6.8±1.8	35.8±3.8	<0.001	<0.001	<0.001

Values are means ± SE (*n* = 14–17). HFD, high fat diet; LFD, low fat diet.

Plasma glucose, insulin as well as plasma leptin levels were all significantly higher in HFD mice when compared to LFD mice. For plasma leptin and insulin we also detected a significant time-induced increase. Additionally, a significant diet * time interaction for insulin was observed, with a larger increase in plasma insulin over time in HFD *vs.* LFD mice.

As a result, HOMA-IR was significantly higher in HFD *vs.* LFD mice and at week 20 *vs.* week 8, indicative of insulin resistance. In addition, a significant diet * time interaction was found, showing a more pronounced increase of HOMA-IR over time in HFD as compared to LFD mice (2.36-fold *vs.* 1.55-fold in HFD *vs.* LFD).

## Discussion

Type 2 diabetes mellitus and (lipid-induced) insulin resistance have been associated with disturbances in skeletal muscle mitochondrial metabolism. In the present study we examined the hypothesis that prolonged consumption of a high-fat diet (HFD) eventually results in more saturated mitochondrial phospholipids, which in turn hampers the mitochondrial oxidative capacity possibly contributing to the occurrence of insulin resistance. To test this hypothesis, we subjected mice to 8-week and 20-week HFD feeding and measured skeletal muscle mitochondrial phospholipid composition, mitochondrial oxidation of carbohydrate- and lipid-derived substrates, muscle mitochondrial density and insulin sensitivity.

We found that skeletal muscle mitochondrial phospholipids of HFD mice contained relatively less MUFA than mitochondrial phospholipids of LFD mice. In line with our hypothesis, the decrease in MUFA was indeed paralleled by an increase in SFA at week 8. Surprisingly however, after 20 weeks of dietary intervention the decrease in MUFA was accompanied by a prominent increase in PUFA rather than an increase in SFA. Despite the increase in SFA in mitochondrial phospholipids at week 8, mitochondrial oxidation of both pyruvate and fatty acids was comparable between LFD and HFD mice. At week 20, we found that maximally coupled (ADP-stimulated) respiration on palmitoyl-CoA tended to be higher in HFD mice *vs.* LFD mice. The maximally uncoupled respiration, indicating the maximal capacity of the electron transport chain on this lipid substrate, was significantly higher in HFD mice after 20 weeks of dietary intervention. Irrespective of these changes in mitochondrial membrane composition and mitochondrial oxidative capacity, the HOMA-IR index indicated that HFD mice were insulin resistant at both 8 and 20 weeks.

Cellular membranes such as the mitochondrial membranes maintain and regulate ionic gradients, potential differences and uptake of substrates such as fatty acyl CoAs [28]. The FA composition and the degree of saturation of these phospholipids are of great importance for fluidity, permeability and thus for proper function of the mitochondrial membrane [28], [29]. In this context, numerous studies have linked the FA composition of skeletal muscle phospholipids and insulin sensitivity. A reduced insulin sensitivity is associated with high amounts of SFA and a low n-3 *vs.* n-6 PUFA ratio [25], [26], [27], [30]. Previously, we demonstrated that a 4-week HFD results in an increased saturation of skeletal muscle phospholipids. This increased saturation was especially evident in phosphatidylcholine and phosphatidylethanolamine [16], which make up more than 70 percent of the mouse skeletal muscle mitochondrial membrane (data not shown). Here, we found that skeletal muscle mitochondrial phospholipids of HFD mice contained less MUFA and more SFA after 8 weeks of dietary intervention, corroborating our previous findings [16]. To our knowledge, we are the first to show that increases in SFA and decreases in the ratio n-3 *vs.* n-6 PUFA are also observed in skeletal muscle mitochondrial membrane phospholipids of HFD-induced insulin resistant mice.

To test whether HFD-induced changes in FA composition of mitochondrial phospholipids lead to an altered mitochondrial oxidative capacity, we performed high-resolution respirometry in isolated skeletal muscle mitochondria using both a carbohydrate- (pyruvate) and a lipid-derived (palmitoyl-CoA+carnitine) substrate.

We observed an increased saturation of the mitochondrial phospholipid composition after 8 weeks of HFD. Because increased levels of SFA in mitochondrial membranes were shown to be associated with decreased activity of mitochondrial enzymes [23] we anticipated an impaired mitochondrial respiratory capacity upon 8 weeks of HFD. Surprisingly however, mitochondrial oxidation of both pyruvate and palmitoyl-CoA+carnitine was not altered upon 8 weeks of HFD.

Interestingly, we found that the increase of n-6 PUFA and the higher unsaturation index in HFD mice after 20 weeks of dietary intervention coincided with an enhanced maximal capacity of the electron transport chain as well as a tendency for an increased ADP-stimulated respiration upon the lipid substrate (Figure 1). An increased desaturation improves membrane fluidity and responsiveness of membrane-bound proteins [24], [29]. Therefore, one could speculate that this increased desaturation of mitochondrial phospholipids contributed to the enhanced mitochondrial oxidation of palmitoyl-CoA. However, the increased desaturation did not improve mitochondrial respiration driven by pyruvate of HFD mice, indicating that it is not the mitochondrial phospholipid composition *per se* that alters mitochondrial respiratory capacity.

It should also be noted that our mitochondrial isolation did not distinguish between intermyofibrillar and subsarcolemmal mitochondria, two mitochondrial pools that can have distinct metabolic characteristics [31]. Furthermore, our *ex vivo* analysis of mitochondrial oxidation represents the maximal capacity of isolated mitochondria under optimal experimental conditions. We cannot fully exclude that one of the two mitochondrial subpopulations or *in vivo* ATP synthesis rate were negatively affected by the dietary interventions. Finally, our mitochondrial oxidation analyses do not allow us to single out the individual maximal capacity of complex II, III and/or IV of the respiratory chain, which could still be hampered by HF feeding.

Taken together, an 8-week HFD did not negatively impact mitochondrial oxidation of a carbohydrate- or a lipid-derived substrate, despite an increased saturation of mitochondrial phospholipids. Prolonged (20 week) HFD feeding even seemed to improve mitochondrial fat oxidative capacity, which was accompanied by an increased unsaturation of the mitochondrial membrane phospholipids.

Besides diminished intrinsic mitochondrial capacity, decreased muscle oxidative capacity can also be caused by reductions in mitochondrial density. Therefore, we determined the effect of our 8-week and 20-week dietary intervention on several markers of mitochondrial density in skeletal muscle. However, none of the assessed markers in the tibialis anterior (TA) muscle supported a reduction in muscle mitochondrial density.

To be able to determine both mitochondrial phospholipid composition and mitochondrial respiration in each individual mouse, we combined all hind limb muscles in order to isolate sufficient amounts of mitochondria. Therefore, we cannot fully exclude the possibility that the different dietary interventions may have differentially affected mitochondria from distinct muscle types. In this context, we have also determined the phospholipid composition of pooled mitochondrial preparations derived from individual muscles (gastrocnemius and quadriceps) obtained in parallel groups of mice (Supporting Information S3). In line with our findings in mitochondria derived from combined hind limb muscles, these experiments showed similar changes in mitochondrial phospholipid composition in response to the dietary interventions (Supporting Information S4). In addition, one could also argue that the TA muscle, used to assess muscle mitochondrial density, might not be a representative muscle for our combined muscle mitochondria. Therefore we also determined the mitochondrial density markers in the contralateral gastrocnemius and quadriceps muscles obtained from the previously mentioned parallel experiments focusing on pooled mitochondria per individual muscle. Although the different muscles responded somewhat differently to the dietary interventions, we did not find evidence for reduced mitochondrial content in any of the muscles studied (Supporting Information S5).

In contrast to our findings, it was previously shown that long-term (16 weeks), but not short-term (4 weeks), high-fat/high-sucrose feeding in mice was associated with a reduced oxygen consumption in permeabilized muscle fibers [12]. These findings were most likely explained by a decrease in mitochondrial density. Along the same line, it was shown that genes involved in mitochondrial biogenesis in skeletal muscle were down-regulated after 3 weeks of high-fat diet in mice [11]. On the other hand, our results are in agreement with other studies suggesting that an HFD does not induce insulin resistance by decreasing skeletal muscle mitochondrial (fat) oxidative capacity [16], [17]. In fact, several other studies suggest that mitochondrial capacity in skeletal muscle is even increased upon the consumption of a high-fat diet in animals [13], [14], [15]. The discrepancies in results concerning the impact of high-fat feeding on skeletal mitochondrial capacity may be (partly) explained by the variability in diets used in literature. In other words, differences in the duration of the dietary intervention as well as the quantity and source of both fat and carbohydrates (e.g. sucrose levels) complicate a direct comparison between these studies.

We found that the 8-week and 20-week HFD induced different changes in the skeletal muscle mitochondrial phospholipid composition Interestingly, negative effects of the HFD on mitochondrial oxidation of pyruvate and/or fatty acids as well as on mitochondrial density were not observed. However, insulin sensitivity was impaired in HFD mice at both 8 and 20 weeks. Thus, HOMA-IR was significantly higher in HFD mice *vs.* LFD mice and this difference was most pronounced at 20 weeks. Moreover, de Wit et al. [32] demonstrated that a 7-week intervention with this specific HFD results in decreased glucose tolerance as assessed by OGTT. As elevated HOMA-IR and impaired glucose tolerance assessed by OGTT do not necessarily reflect skeletal muscle insulin resistance we also included data from hyperinsulinemic-euglycemic clamps upon 5 weeks of dietary intervention. This experiment (Supporting Information S6) clearly indicates that peripheral insulin-stimulated glucose uptake, which is primarily accounted for by skeletal muscle, is markedly reduced after 5 weeks of HFD feeding. More importantly, we also assessed the level of IRS-1 serine phosporylation at Ser^307^ in skeletal muscle tissue by Western blotting and found a significantly increased signal after 8 weeks of HFD feeding, indicating muscle insulin resistance (Supporting Information S7) [33]. Although our findings do not support a causal role for a reduced mitochondrial oxidative capacity in causing muscle insulin resistance, it remains possible however, that an HFD-induced increase in skeletal muscle mitochondrial ROS production contributes to the observed insulin resistance. In this context, we determined 4-HNE protein adducts as a marker for oxidative stress in the TA, gastrocnemius and quadriceps muscle. We did not find evidence for increased oxidative stress upon the HFD in any of the studied muscles (Supporting Information S8).

In conclusion, our results show that long-term - in contrast to mid-term - consumption of a high-fat diet does not increase the saturation of mouse muscle mitochondrial phospholipids. Despite the development of insulin resistance, skeletal muscle mitochondrial density and mitochondrial oxidation of pyruvate and fatty acids (palmitoyl-CoA + carnitine) were not negatively affected upon both mid- and long-term HFD feeding. These findings do not support the concept that changes in the saturation of skeletal muscle mitochondrial phospholipids contribute to a decrease in mitochondrial fat oxidative capacity, which may underlie the occurrence of (muscle) insulin resistance upon HFD feeding.

## Materials and Methods

### Ethics statement

The experimental protocol was approved by the Local Committees for Care and Use of Laboratory Animals at Maastricht University (approval number 2008-074) and Wageningen University (approval number 2008-033b) and complied with the principles of laboratory animal care.

### Animals and diets

Male C57BL/6J mice were obtained from Harlan (Horst, the Netherlands). At 9 weeks of age mice were fed the run-in diet consisting of the low fat diet (10 kcal%; LFD) for 3 weeks. Following this run-in period mice were randomly assigned to the LFD or the HFD (45 kcal %) for 8 or 20 weeks. Both diets contained fat in the form of palm oil (based on D12450B and D12451; Research Diet Services, Wijk bij Duurstede, the Netherlands) as described [32]. The FA composition of palm oil is shown in Supporting Information S9. Diets and tap water were provided *ad libitum*. Food intake and body mass were recorded weekly. During week 7 and 19 of dietary intervention faecal samples were collected for 1 week. To calculate net energy intake during this last week of the dietary intervention, faecal samples were freeze-dried and, together with samples from the diet, analyzed for gross energy content using adiabatic bomb calorimetry (Ika-calorimeter system C4000, Heitersheim, Germany).

### Tissue collection and mitochondrial isolation

Mice (*n* = 8 per time point) were sedated by a mixture of 79% CO_2_ and 21% O_2_ and killed by decapitation. Muscle tissue of both hind limbs was rapidly dissected and placed into ice-cold mitochondrial isolation buffer (∼10 ml; 100 mM sucrose, 50 mM KCl, 20 mM K^+^-TES, 1 mM EDTA and 0.2% (w/v) BSA). Skeletal muscle mitochondria were isolated as described [34]. Freshly isolated mitochondria were used for respiration experiments. The tibialis anterior (TA) muscle was dissected separately, snap-frozen in liquid nitrogen en stored at −80°C for further analysis.

### High-resolution respirometry

Mitochondrial protein concentrations were measured using fluorescamine (Fluram®, Fluka, Zwijndrecht, the Netherlands) with BSA as a standard [35]. The freshly isolated mitochondria were immediately used for respiration experiments. Mitochondrial respiration rates were measured as described [17], [34], [36] using a two-chamber Oxygraph (Oroboros® Instruments, Innsbruck, Austria). Pyruvate (5 mM, in the presence of 3 mM malate) was used as carbohydrate-derived substrate and 2 mM carnitine + 50 µM palmitoyl-CoA was used as FA substrate.

Remaining mitochondria were stored at −80°C for analysis of the FA composition of mitochondrial phospholipids.

### Fatty acid composition of mitochondrial phospholipids

Phospholipids were isolated from frozen mitochondrial samples by thin-layer chromatography, subsequently hydrolyzed and methylated into their corresponding FA methyl esters. These FA methyl esters were separated and quantified by gas chromatography as described [37]. The unsaturation index was calculated according to the following formula [38]: Unsaturation index = Σ % of unsaturated FA * number of double bounds of each unsaturated FA.

### Measures of mitochondrial density

As parameters for mitochondrial density we measured mitochondrial DNA copy number, protein levels of structural components of the five complexes of the respiratory chain and activity levels of two mitochondrial enzymes in the TA muscle.

#### Mitochondrial DNA copy number

Total DNA was isolated from the TA muscle using the DNeasy Blood & Tissue Kit (Qiagen, Venlo, the Netherlands) according to the manufacturer's instructions. The relative mitochondrial copy number was measured with minor adaptations as described [39]. Briefly, the amount of nuclear DNA (nDNA) and mitochondrial DNA (mtDNA) from 1.25 ng total DNA were determined by qPCR with primers specific for mtDNA (Cytb) and nDNA (Rn18S). The relative mitochondrial copy number is presented as the ratio of mtDNA to nDNA.

#### Western blotting of protein levels of mitochondrial respiration chain complexes

Protein levels of subunits of complexes of the mitochondrial respiratory chain were detected in TA muscle protein extracts (*n* = 6) using a mixture of monoclonal OXPHOS antibodies (MitoSciences, Oregon, USA) directed to the ND6 subunit of complex I, the 30 kDa Ip subunit of complex II, the 47 kDa core protein 2 of complex III, subunit II of cytochrome C oxidase (COXII) and the alpha subunit of the F1F0 ATP synthase (complex V) as described previously [16]. Porin protein levels were measured as described previously [40].

#### Activity of mitochondrial enzymes

Activity levels of the mitochondrial enzymes β-hydroxyacyl-CoA dehydrogenase (HAD; β-oxidation) and citrate synthase (CS; TCA cycle) were determined in TA homogenates (*n* = 6–8) as described by Den Hoed et al [41].

### Plasma parameters and glucose homeostasis

Blood was obtained by orbital puncture and collected in EDTA-containing tubes (Sarstedt AG&CO, Nümbrecht, Germany). Plasma was obtained after centrifugation at 11000×g for 10 min and stored at −80°C. Plasma insulin levels were determined by the Insulin (Mouse) Ultrasensitive EIA (Alpco Diagnostics, Salem, NH, USA) and plasma leptin levels were measured with the Quantikine Mouse Leptin Immunoassay (R&D systems, Minneapolis, MN, USA). Plasma glucose levels were measured with the Accu-Chek (Roche Diagnostics, Almere, the Netherlands) after a 6-hour fast. HOMA-IR was calculated from fasting glucose and insulin levels (fasting glucose * fasting insulin/22.5).

### Statistical analyses

All data are expressed as means ± SEM. Statistical analysis was performed using SPSS for Windows version 15.0 software (SPSS Inc., Chicago, IL, USA). Two-way ANOVA with univariate analysis of variance was performed to analyze effects of diet (HFD *vs.* LFD), time (week 20 *vs.* week 8) and interaction (diet * time). Statistical significance was set at *p*<0.05.

## Supporting Information

Supporting Information S1 Absolute amounts of SFA, MUFA, PUFA, n-3 PUFA and n-6 PUFA in mitochondrial phospholipids from hind limb muscles. Results are expressed as umol/ml per mg protein. HFD, high fat diet; LFD, low fat diet; MUFA, mono-unsaturated fatty acids; PUFA, poly-uunsaturated fatty acids; SFA, saturated fatty acids; UI, unsaturation index.
(DOC)

Supporting Information S2 Western blotting of subunits of the five complexes of the respiration chain (OxPhos) in TA muscle of mice fed a LFD or HFD for 8 or 20 weeks, respectively. Shown are representative examples of equal amounts of total TA muscle protein (*n* = 6). OxPhos normalized for Gapdh signal was used as marker for mitochondrial density. OxPhos normalized for porin was used to study intramitochondrial changes in OxPhos. HFD, high fat diet; LFD, low fat diet; TA, tibialis anterior.
(DOC)

Supporting Information S3 Mitochondrial phospholipid composition in individual muscle types.
(DOC)

Supporting Information S4 Relative amounts of most abundant fatty acids, the ratio n-3 vs. n-6 and the unsaturation index in mitochondrial phospholipids from gastrocenmius and quadriceps muscles. Within each muscle type mitochondria were pooled per diet group. Therefore, data lack biological variation and statistics could not be performed. As such, no standard errors are shown. HFD, high fat diet; LFD, low fat diet; MUFA, mono-unsaturated fatty acids; PUFA, poly-uunsaturated fatty acids; SFA, saturated fatty acids; UI, unsaturation index.
(DOC)

Supporting Information S5 Parameters for mitochondrial density in quadriceps and gastrocnemius muscles. Parameters in the quadriceps and gastrocnemius muscle in LFD mice and HFD mice at 8 weeks and 20 weeks. Values are means ± SE (n = 15–17 for mitochondrial DNA copy number; n = 6 for sum of the 5 complexes of the respiratory chain and n = 6–7 for CS and HAD activity). CS, citrate synthase; HAD, β-hydroxyacyl-CoA dehydrogenase; HFD, high fat diet; LFD, low fat diet; TA, tibialis anterior.
(DOC)

Supporting Information S6 A hyperinsulinemic euglycemic clamp was performed in mice fed the LFD and HFD for 5 weeks as described previously [4]. The steady-state glucose infusion rate (GIR) was significantly lower in 5-week HFD mice than LFD mice (A). Plasma glucose levels did not differ between HFD mice and LFD mice during the clamp (B). Peripheral insulin sensitivity is expressed as the rate of disappearance (Rd) during the basal and hyperinsulinemic period. Insulin significantly increased the uptake of glucose by peripheral tissues in LFD mice, but not in HFD mice (C). Peripheral insulin sensitivity is expressed as the percentage of increase of glucose Rd during the hyperinsulinemic state compared to basal. The ability of insulin to stimulate the rate of disappearance of glucose was significantly lower in HFD mice than in LFD mice. Hepatic insulin sensitivity was expressed as the percentage of repression of hepatic glucose production (Ra) during the hyperinsulinemic state compared to basal. The ability of insulin to inhibit hepatic glucose production in HFD mice was similar to LFD mice suggesting that the liver was still insulin sensitive (D). LFD mice and HFD mice are indicated in black and white, respectively. Values are means ± SE (N = 5–8).*; p<0.05 HFD compared to LFD, $; p<0.05 hyper compared to basal. HFD, high fat diet; LFD, low fat diet.
(DOC)

Supporting Information S7 IRS-1 serine phosphorylation in LFD (black bar) and HFD (white bar) mice after 8 weeks of dietary intervention. Briefly, equal amounts of muscle membrane protein fractions were loaded on SDS-PAGE. After Western blotting, membranes were incubated with an antibody detecting IRS-1 phosphorylation at Ser^307^ (#2381S, Cell Signaling Technology, Bioké, Leiden, The Netherlands). After incubation with the appropriate secondary IRDye680-labeled antibody (Licor, Westburg, Leusden, the Netherlands), the specific IRS-1 bands were detected and analyzed using the Odyssey near Infrared Scanner (Licor). Values are mean ± SE (n = 4). * p<0.05, assessed by independent samples t-test. The increased IRS-1 serine phosphorylation indicates the presence of muscle insulin resistance.
(DOC)

Supporting Information S8 Quantification of 4-HNE protein adducts in the quadriceps, gastrocnemius and TA muscle of mice fed an LFD or HFD for 8 and 20 weeks, respectively. Protein adducts of the lipid peroxidation byproduct 4-hydroxynonenal (4-HNE) were determined as marker of lipid peroxidation. Western blotting was performed as described [5]. We did not detect an increase in oxidative stress upon 8 or 20 weeks of HFD in any of the muscles studied. Thus, neither diet, nor time significantly changed the level of 4-HNE protein adducts in the quadriceps (A) as well as the TA muscle (C). In the gastrocnemius (B) we observed a significant diet * time effect with an increase in 4-HNE protein adducts over time in LFD mice, whereas a decrease over time was seen in HFD mice (LFD: 0.99 *vs.* 1.13 and HFD: 0.99 *vs.* 0.92 in 8-week *vs.* 20-week). Black bars and white bars represent LFD mice and HFD mice, respectively. Values (arbitrary units) are means ± SE (n = 6). D*T, significant diet * time effect with *p*<0.05; HFD, high fat diet; LFD, low fat diet; TA, tibialis anterior.
(DOC)

Supporting Information S9 Fatty acid composition of palm oil.
(DOC)
