# Supplementary material for: High Fat Diet-Induced Changes in Mouse Muscle Mitochondrial Phospholipids Do Not Impair Mitochondrial Respiration Despite Insulin Resistance
Source: PLoS One. 2011 Nov 28;6(11):e27274. doi: 10.1371/journal.pone.0027274 (PMC3225362; doi:10.1371/journal.pone.0027274)
Supplement: Supporting Information S1 — Absolute amounts of SFA, MUFA, PUFA, n-3 PUFA and n-6 PUFA in mitochondrial phospholipids from hind limb muscles. Results are expressed as umol/ml per mg protein. HFD, high fat diet; LFD, low fat diet; MUFA, mono-unsaturated fatty acids; PUFA, poly-uunsaturated fatty acids; SFA, saturated fatty acids; UI, unsaturation index. (DOC) [file pone.0027274.s001.doc]

# Supporting Information 1

## High fat diet-induced changes in mouse muscle mitochondrial phospholipid composition and function are unrelated to insulin resistance

Joris Hoeks^1,*^, Janneke de Wilde^1,2*^, Martijn F.M. Hulshof^1,2^,Sjoerd .A.A. van den Berg^2,3^, Gert Schaart^4^, Ko Willems van Dijk^1,3,5^, Egbert Smit^1,2^, Edwin.C.M. Mariman^1,2^

* both authors contributed equally

^1^NUTRIM School for Nutrition, Toxicology and Metabolism, Department of Human Biology, Maastricht University Medical Center^+^, Maastricht, the Netherlands; ^2^Top Institute Food and Nutrition, Nutrigenomics Consortium, Wageningen, the Netherlands; ^3^Department of Human Genetics, University Medical Center Leiden, Leiden, the Netherlands; ^4^NUTRIM School for Nutrition, Toxicology and Metabolism, Department of Human Movement Sciences, Maastricht University Medical Center^+^, Maastricht, the Netherlands; ^5^Department of Internal Medicine, University Medical Center Leiden, Leiden, the Netherlands

Supporting Information 1: Absolute amounts of SFA, MUFA, PUFA, n-3 PUFA and n-6 PUFA in mitochondrial phospholipids from hind limb muscles

|  | Week 8 | Week 8 | Week 20 | Week 20 |
| --- | --- | --- | --- | --- |
|  | LFD | HFD | LFD | HFD |
| SFA | 0.132 ± 0.007 | 0.142 ± 0.006 | 0.134 ± 0.010 | 0.150 ± 0.004 |
| 16:0 | 0.080 ± 0.005 | 0.090 ± 0.004 | 0.083 ± 0.007 | 0.096 ± 0.003 |
| 18:0 | 0.048 ± 0.003 | 0.048 ± 0.002 | 0.047 ± 0.003 | 0.050 ± 0.002 |
| MUFA | 0.050 ± 0.003 | 0.037 ± 0.002 | 0.052 ± 0.004 | 0.041 ± 0.001 |
| 16:1n7 | 0.010 ± 0.001 | 0.004 ± 0.000 | 0.011 ± 0.001 | 0.005 ± 0.000 |
| 18:1n7 | 0.017 ± 0.001 | 0.012 ± 0.001 | 0.016 ± 0.001 | 0.012 ± 0.000 |
| 18:1n9 | 0.020 ± 0.001 | 0.019 ± 0.001 | 0.022 ± 0.002 | 0.022 ± 0.001 |
| PUFA | 0.150 ± 0.009 | 0.153 ± 0.007 | 0.148 ± 0.011 | 0.171 ± 0.005 |
| n-3 PUFA | 0.057 ± 0.003 | 0.053 ± 0.003 | 0.058 ± 0.004 | 0.059 ± 0.002 |
| 22:6n3 | 0.052 ± 0.003 | 0.048 ± 0.002 | 0.053 ± 0.004 | 0.054 ± 0.002 |
| n-6 PUFA | 0.091 ± 0.005 | 0.098 ± 0.005 | 0.089 ± 0.007 | 0.111 ± 0.003 |
| 18:2n6 | 0.033 ± 0.002 | 0.034 ± 0.002 | 0.031 ± 0.003 | 0.040 ± 0.002 |
| 20:4n6 | 0.039 ± 0.002 | 0.041 ± 0.002 | 0.038 ± 0.003 | 0.044 ± 0.002 |
| 22:5n6 | 0.011 ± 0.000 | 0.015 ± 0.001 | 0.012 ± 0.001 | 0.018 ± 0.001 |

Results are expressed as umol/ml per mg protein. HFD, high fat diet; LFD, low fat diet; MUFA, mono-unsaturated fatty acids; PUFA, poly-uunsaturated fatty acids; SFA, saturated fatty acids; UI, unsaturation index
