# Supplementary material for: High Fat Diet-Induced Changes in Mouse Muscle Mitochondrial Phospholipids Do Not Impair Mitochondrial Respiration Despite Insulin Resistance
Source: PLoS One. 2011 Nov 28;6(11):e27274. doi: 10.1371/journal.pone.0027274 (PMC3225362; doi:10.1371/journal.pone.0027274)
Supplement: Supporting Information S2 — Western blotting of subunits of the five complexes of the respiration chain (OxPhos) in TA muscle of mice fed a LFD or HFD for 8 or 20 weeks, respectively. Shown are representative examples of equal amounts of total TA muscle protein (n = 6). OxPhos normalized for Gapdh signal was used as marker for mitochondrial density. OxPhos normalized for porin was used to study intramitochondrial changes in OxPhos. HFD, high fat diet; LFD, low fat diet; TA, tibialis anterior. (DOC) [file pone.0027274.s002.doc]

# Supporting Information 2

## High fat diet-induced changes in mouse muscle mitochondrial phospholipid composition and function are unrelated to insulin resistance

Joris Hoeks^1,*^, Janneke de Wilde^1,2*^, Martijn F.M. Hulshof^1,2^,Sjoerd .A.A. van den Berg^2,3^, Gert Schaart^4^, Ko Willems van Dijk^1,3,5^, Egbert Smit^1,2^, Edwin.C.M. Mariman^1,2^

* both authors contributed equally

^1^NUTRIM School for Nutrition, Toxicology and Metabolism, Department of Human Biology, Maastricht University Medical Center^+^, Maastricht, the Netherlands; ^2^Top Institute Food and Nutrition, Nutrigenomics Consortium, Wageningen, the Netherlands; ^3^Department of Human Genetics, University Medical Center Leiden, Leiden, the Netherlands; ^4^NUTRIM School for Nutrition, Toxicology and Metabolism, Department of Human Movement Sciences, Maastricht University Medical Center^+^, Maastricht, the Netherlands; ^5^Department of Internal Medicine, University Medical Center Leiden, Leiden, the Netherlands

| 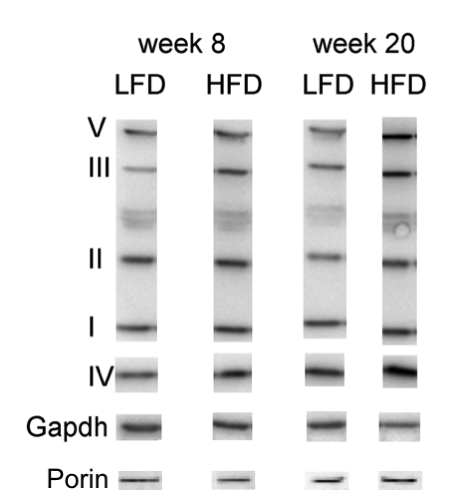 |
| --- |

Supporting Information 2: Western blotting of subunits of the five complexes of the respiration chain (OxPhos) in TA muscle of mice fed a LFD or HFD for 8 or 20 weeks, respectively.

Shown are representative examples of equal amounts of total TA muscle protein (n = 6). OxPhos normalized for Gapdh signal was used as marker for mitochondrial density. OxPhos normalized for porin was used to study intramitochondrial changes in OxPhos. HFD, high fat diet; LFD, low fat diet; TA, tibialis anterior.
