# Supplementary material for: High Fat Diet-Induced Changes in Mouse Muscle Mitochondrial Phospholipids Do Not Impair Mitochondrial Respiration Despite Insulin Resistance
Source: PLoS One. 2011 Nov 28;6(11):e27274. doi: 10.1371/journal.pone.0027274 (PMC3225362; doi:10.1371/journal.pone.0027274)
Supplement: Supporting Information S3 — Mitochondrial phospholipid composition in individual muscle types. (DOC) [file pone.0027274.s003.doc]

# Supporting Information 3

## High fat diet-induced changes in mouse muscle mitochondrial phospholipid composition and function are unrelated to insulin resistance

Joris Hoeks^1,*^, Janneke de Wilde^1,2*^, Martijn F.M. Hulshof^1,2^,Sjoerd .A.A. van den Berg^2,3^, Gert Schaart^4^, Ko Willems van Dijk^1,3,5^, Egbert Smit^1,2^, Edwin.C.M. Mariman^1,2^

* both authors contributed equally

^1^NUTRIM School for Nutrition, Toxicology and Metabolism, Department of Human Biology, Maastricht University Medical Center^+^, Maastricht, the Netherlands; ^2^Top Institute Food and Nutrition, Nutrigenomics Consortium, Wageningen, the Netherlands; ^3^Department of Human Genetics, University Medical Center Leiden, Leiden, the Netherlands; ^4^NUTRIM School for Nutrition, Toxicology and Metabolism, Department of Human Movement Sciences, Maastricht University Medical Center^+^, Maastricht, the Netherlands; ^5^Department of Internal Medicine, University Medical Center Leiden, Leiden, the Netherlands

Supporting Information 3: Mitochondrial phospholipid composition in individual muscle types

To assess the FA composition of mitochondrial phospholipids of gastrocnemius and quadriceps muscles separately, mice (week 8: n = 15; week 20: n = 17) were killed as described [1]. Muscles from the left hind leg were placed into ice-cold buffer containing 20 mM MOPS, 100 mM KCl and 1mM EGTA (MKE). Mitochondria were isolated as described in Frezza et al. [2] with minor adaptations. Muscles were freed of fat, minced, suspended in MKE containing phosphatase inhibitors (Sigma, Zwijndrecht, the Netherlands, MKEP) and centrifuged (1000 x g for 30 s at 4 °C). Pellets were suspended in ~ 1 ml MKEP with trypsin (0.5 mg/ml; Sigma), incubated for 3 min on ice and centrifuged. This step was repeated once with 30 min incubation. Per ml MKEP 250 µl quencher solution was added (50 mg/ml FA-free BSA, phosphatase inhibitors, proteinase inhibitors and 200 mM phenylmethanesulfonyl (PMSF, Sigma)) followed by centrifugation. Next, pellets were suspended in MKEP containing proteinase inhibitors and 200 mM PMSF (MKE3P) and centrifuged. Muscle pellets were suspended in 1.5 ml MKE3P, mechanically homogenized, centrifuged at 800 x g for 5 min at 4 °C and supernatant was collected. This step was repeated twice. The collected supernatants were centrifuged at 15000 x g for 10 min at 4 °C, the mitochondrial pellet was dissolved in 1-2 ml MKE3P, loaded on 2-step percoll gradient and centrifuged as described [3]. Mitochondria were collected by syringe aspiration, washed with MKE3P and centrifuged at 15000 x g for 10 min at 4 °C. The mitochondrial pellet was dissolved in 250 µl MKE. An aliquot of 50 µl was used to measure the protein concentration with the BCA protein assay kit (Pierce, Etten-Leur, the Netherlands). Mitochondria were stored at – 80 °C for further analysis.

## References

1. de Wilde, J., et al., The embryonic genes Dkk3, Hoxd8, Hoxd9 and Tbx1 identify muscle types in a diet-independent and fiber-type unrelated way. BMC Genomics, 2010. **11**: p. 176-187.

2. Frezza, C., S. Cipolat, and L. Scorrano, Organelle isolation: functional mitochondria from mouse liver, muscle and cultured fibroblasts. Nat Protoc, 2007. **2**(2): p. 287-95.

3. Mootha, V.K., et al., Identification of a gene causing human cytochrome c oxidase deficiency by integrative genomics. Proc Natl Acad Sci U S A, 2003. **100**(2): p. 605-10.
