# Supplementary material for: High Fat Diet-Induced Changes in Mouse Muscle Mitochondrial Phospholipids Do Not Impair Mitochondrial Respiration Despite Insulin Resistance
Source: PLoS One. 2011 Nov 28;6(11):e27274. doi: 10.1371/journal.pone.0027274 (PMC3225362; doi:10.1371/journal.pone.0027274)
Supplement: Supporting Information S4 — Relative amounts of most abundant fatty acids, the ratio n-3 vs. n-6 and the unsaturation index in mitochondrial phospholipids from gastrocenmius and quadriceps muscles. Within each muscle type mitochondria were pooled per diet group. Therefore, data lack biological variation and statistics could not be performed. As such, no standard errors are shown. HFD, high fat diet; LFD, low fat diet; MUFA, mono-unsaturated fatty acids; PUFA, poly-uunsaturated fatty acids; SFA, saturated fatty acids; UI, unsaturation index. (DOC) [file pone.0027274.s004.doc]

# Supporting Information 4

## High fat diet-induced changes in mouse muscle mitochondrial phospholipid composition and function are unrelated to insulin resistance

Joris Hoeks^1,*^, Janneke de Wilde^1,2*^, Martijn F.M. Hulshof^1,2^,Sjoerd .A.A. van den Berg^2,3^, Gert Schaart^4^, Ko Willems van Dijk^1,3,5^, Egbert Smit^1,2^, Edwin.C.M. Mariman^1,2^

* both authors contributed equally

^1^NUTRIM School for Nutrition, Toxicology and Metabolism, Department of Human Biology, Maastricht University Medical Center^+^, Maastricht, the Netherlands; ^2^Top Institute Food and Nutrition, Nutrigenomics Consortium, Wageningen, the Netherlands; ^3^Department of Human Genetics, University Medical Center Leiden, Leiden, the Netherlands; ^4^NUTRIM School for Nutrition, Toxicology and Metabolism, Department of Human Movement Sciences, Maastricht University Medical Center^+^, Maastricht, the Netherlands; ^5^Department of Internal Medicine, University Medical Center Leiden, Leiden, the Netherlands

Supporting Information 4: Relative amounts of most abundant fatty acids, the ratio n-3 vs. n-6 and the unsaturation index in mitochondrial phospholipids from gastrocenmius and quadriceps muscles

|  | Gastrocnemius | | Gastrocnemius | | Quadriceps | | Quadriceps | |
| --- | --- | --- | --- | --- | --- | --- | --- | --- |
|  | Week 8 | Week 8 | Week 20 | Week 20 | Week 8 | Week 8 | Week 20 | Week 20 |
|  | LFD | HFD | LFD | HFD | LFD | HFD | LFD | HFD |
| SFA (%) | 40.4 | 43.2 | 40.0 | 43.5 | 40.5 | 42.1 | 39.7 | 42.3 |
| 16:0 (%) | 23.9 | 26.7 | 24.9 | 27.8 | 24.3 | 25.8 | 24.0 | 26.9 |
| 18:0 (%) | 15.3 | 15.4 | 13.8 | 14.6 | 15.1 | 15.4 | 14.5 | 14.5 |
| MUFA (%) | 14.6 | 11.1 | 15.1 | 11.9 | 14.4 | 10.4 | 14.6 | 11.2 |
| 16:1n7 (%) | 3.2 | 1.4 | 3.5 | 1.3 | 3.2 | 1.4 | 3.2 | 1.3 |
| 18:1n7 (%) | 4.7 | 3.5 | 4.9 | 3.7 | 4.5 | 3.3 | 4.5 | 3.7 |
| 18:1n7 (%) | 4.7 | 3.5 | 4.9 | 3.7 | 4.5 | 3.3 | 4.5 | 3.7 |
| PUFA (%) | 45.0 | 45.6 | 44.8 | 44.4 | 45.0 | 47.4 | 45.8 | 46.4 |
| n-3 PUFA (%) | 17.3 | 16.7 | 20.1 | 16.7 | 17.2 | 15.4 | 18.4 | 15.5 |
| 22:6n3 (%) | 15.6 | 15.1 | 18.6 | 15.3 | 15.5 | 13.9 | 17.0 | 14.2 |
| n-6 PUFA (%) | 27.3 | 28.5 | 24.3 | 27.3 | 27.4 | 31.6 | 26.9 | 30.5 |
| 18:2n6 (%) | 11.0 | 9.8 | 8.9 | 9.3 | 11.1 | 12.2 | 10.7 | 11.3 |
| 20:4n6 (%) | 11.0 | 11.0 | 9.8 | 10.4 | 10.9 | 12.3 | 11.0 | 12.3 |
| 22:5n6 (%) | 2.9 | 4.8 | 3.4 | 4.7 | 2.9 | 4.4 | 3.0 | 4.2 |
| n-3 vs. n-6 PUFA | 0.63 | 0.59 | 0.83 | 0.61 | 0.63 | 0.49 | 0.68 | 0.51 |
| UI | 205.3 | 207.6 | 216.0 | 205.0 | 204.7 | 206.9 | 212.1 | 205.8 |

Within each muscle type mitochondria were pooled per diet group. Therefore, data lack biological variation and statistics could not be performed. As such, no standard errors are shown. HFD, high fat diet; LFD, low fat diet; MUFA, mono-unsaturated fatty acids; PUFA, poly-uunsaturated fatty acids; SFA, saturated fatty acids; UI, unsaturation index
