# Supplementary material for: High Fat Diet-Induced Changes in Mouse Muscle Mitochondrial Phospholipids Do Not Impair Mitochondrial Respiration Despite Insulin Resistance
Source: PLoS One. 2011 Nov 28;6(11):e27274. doi: 10.1371/journal.pone.0027274 (PMC3225362; doi:10.1371/journal.pone.0027274)
Supplement: Supporting Information S5 — Parameters for mitochondrial density in quadriceps and gastrocnemius muscles. Parameters in the quadriceps and gastrocnemius muscle in LFD mice and HFD mice at 8 weeks and 20 weeks. Values are means ± SE (n = 15–17 for mitochondrial DNA copy number; n = 6 for sum of the 5 complexes of the respiratory chain and n = 6–7 for CS and HAD activity). CS, citrate synthase; HAD, β-hydroxyacyl-CoA dehydrogenase; HFD, high fat diet; LFD, low fat diet; TA, tibialis anterior. (DOC) [file pone.0027274.s005.doc]

# Supporting Information 5

## High fat diet-induced changes in mouse muscle mitochondrial phospholipid composition and function are unrelated to insulin resistance

Joris Hoeks^1,*^, Janneke de Wilde^1,2*^, Martijn F.M. Hulshof^1,2^,Sjoerd .A.A. van den Berg^2,3^, Gert Schaart^4^, Ko Willems van Dijk^1,3,5^, Egbert Smit^1,2^, Edwin.C.M. Mariman^1,2^

* both authors contributed equally

^1^NUTRIM School for Nutrition, Toxicology and Metabolism, Department of Human Biology, Maastricht University Medical Center^+^, Maastricht, the Netherlands; ^2^Top Institute Food and Nutrition, Nutrigenomics Consortium, Wageningen, the Netherlands; ^3^Department of Human Genetics, University Medical Center Leiden, Leiden, the Netherlands; ^4^NUTRIM School for Nutrition, Toxicology and Metabolism, Department of Human Movement Sciences, Maastricht University Medical Center^+^, Maastricht, the Netherlands; ^5^Department of Internal Medicine, University Medical Center Leiden, Leiden, the Netherlands

Supporting Information 5: Parameters for mitochondrial density in quadriceps and gastrocnemius muscles

|  | 8 weeks | 8 weeks | 20 weeks | 20 weeks | P value | P value | | P value |
| --- | --- | --- | --- | --- | --- | --- | --- | --- |
| Quadriceps: | LFD | HFD | LFD | HFD | diet | | time | diet * time |
| Mitochondrial DNA copy number (AU) | 1.54 ± 0.32 | 1.63 ± 0.34 | 1.47 ± 0.27 | 1.70 ± 0.28 | 0.599 | | 0.988 | 0.818 |
| Sum of the 5 complexes of respiratory chain (AU) | 3.01 ± 0.46 | 2.42 ± 0.50 | 2.75 ± 0.54 | 1.97 ± 0.24 | 0.139 | | 0.434 | 0.840 |
| CS (μmol/min/g protein) | 21.5 ± 3.0 | 24.1 ± 3.5 | 28.3 ± 2.0 | 29.3 ± 1.9 | 0.538 | | 0.045 | 0.768 |
| HAD (μmol/min/g protein) | 5.7 ± 1.0 | 5.4 ± 0.9 | 10.0 ± 1.4 | 13.2 ± 1.9 | 0.298 | | < 0.001 | 0.208 |
| Gastrocnemius: |  |  |  |  |  | |  |  |
| Mitochondrial DNA copy number (AU) | 1.01 ± 0.20 | 1.18 ± 0.16 | 1.52 ± 0.27 | 1.35 ± 0.27 | 1.000 | | 0.163 | 0.495 |
| Sum of the 5 complexes of respiratory chain (AU) | 1.42 ± 0.09 | 1.97 ± 0.20 | 1.93 ± 0.28 | 2.18 ± 0.14 | 0.050 | | 0.071 | 0.444 |
| CS (μmol/min/g protein) | 27.6 ± 2.2 | 23.7 ± 3.5 | 28.2 ± 3.0 | 33.4 ± 1.6 | 0.821 | | 0.073 | 0.114 |
| HAD (μmol/min/g protein) | 13.3 ± 2.3 | 6.9 ± 1.2 | 10.0 ± 1.6 | 15.3 ± 2.3 | 0.787 | | 0.213 | 0.007 |

Parameters in the quadriceps and gastrocnemius muscle in LFD mice and HFD mice at 8 weeks and 20 weeks. Values are means ± SE (n = 15-17 for mitochondrial DNA copy number; n = 6 for sum of the 5 complexes of the respiratory chain and n = 6-7 for CS and HAD activity). CS, citrate synthase; HAD, β-hydroxyacyl-CoA dehydrogenase; HFD, high fat diet; LFD, low fat diet; TA, tibialis anterior.

## 
