# Supplementary material for: High Fat Diet-Induced Changes in Mouse Muscle Mitochondrial Phospholipids Do Not Impair Mitochondrial Respiration Despite Insulin Resistance
Source: PLoS One. 2011 Nov 28;6(11):e27274. doi: 10.1371/journal.pone.0027274 (PMC3225362; doi:10.1371/journal.pone.0027274)
Supplement: Supporting Information S6 — A hyperinsulinemic euglycemic clamp was performed in mice fed the LFD and HFD for 5 weeks as described previously [4]. The steady-state glucose infusion rate (GIR) was significantly lower in 5-week HFD mice than LFD mice (A). Plasma glucose levels did not differ between HFD mice and LFD mice during the clamp (B). Peripheral insulin sensitivity is expressed as the rate of disappearance (Rd) during the basal and hyperinsulinemic period. Insulin significantly increased the uptake of glucose by peripheral tissues in LFD mice, but not in HFD mice (C). Peripheral insulin sensitivity is expressed as the percentage of increase of glucose Rd during the hyperinsulinemic state compared to basal. The ability of insulin to stimulate the rate of disappearance of glucose was significantly lower in HFD mice than in LFD mice. Hepatic insulin sensitivity was expressed as the percentage of repression of hepatic glucose production (Ra) during the hyperinsulinemic state compared to basal. The ability of insulin to inhibit hepatic glucose production in HFD mice was similar to LFD mice suggesting that the liver was still insulin sensitive (D). LFD mice and HFD mice are indicated in black and white, respectively. Values are means ± SE (N = 5–8).*; p<0.05 HFD compared to LFD, $; p<0.05 hyper compared to basal. HFD, high fat diet; LFD, low fat diet. (DOC) [file pone.0027274.s006.doc]

# Supporting Information 6

## High fat diet-induced changes in mouse muscle mitochondrial phospholipid composition and function are unrelated to insulin resistance

Joris Hoeks^1,*^, Janneke de Wilde^1,2*^, Martijn F.M. Hulshof^1,2^,Sjoerd .A.A. van den Berg^2,3^, Gert Schaart^4^, Ko Willems van Dijk^1,3,5^, Egbert Smit^1,2^, Edwin.C.M. Mariman^1,2^

* both authors contributed equally

^1^NUTRIM School for Nutrition, Toxicology and Metabolism, Department of Human Biology, Maastricht University Medical Center^+^, Maastricht, the Netherlands; ^2^Top Institute Food and Nutrition, Nutrigenomics Consortium, Wageningen, the Netherlands; ^3^Department of Human Genetics, University Medical Center Leiden, Leiden, the Netherlands; ^4^NUTRIM School for Nutrition, Toxicology and Metabolism, Department of Human Movement Sciences, Maastricht University Medical Center^+^, Maastricht, the Netherlands; ^5^Department of Internal Medicine, University Medical Center Leiden, Leiden, the Netherlands

|  |
| --- |
| Supporting Information 6: A hyperinsulinemic euglycemic clamp was performed in mice fed the LFD and HFD for 5 weeks as described previously [1].  The steady-state glucose infusion rate (GIR) was significantly lower in 5-week HFD mice than LFD mice (A). Plasma glucose levels did not differ between HFD mice and LFD mice during the clamp (B). Peripheral insulin sensitivity is expressed as the rate of disappearance (Rd) during the basal and hyperinsulinemic period. Insulin significantly increased the uptake of glucose by peripheral tissues in LFD mice, but not in HFD mice (C). Peripheral insulin sensitivity is expressed as the percentage of increase of glucose Rd during the hyperinsulinemic state compared to basal. The ability of insulin to stimulate the rate of disappearance of glucose was significantly lower in HFD mice than in LFD mice. Hepatic insulin sensitivity was expressed as the percentage of repression of hepatic glucose production (Ra) during the hyperinsulinemic state compared to basal. The ability of insulin to inhibit hepatic glucose production in HFD mice was similar to LFD mice suggesting that the liver was still insulin sensitive (D).  LFD mice and HFD mice are indicated in black and white, respectively. Values are means ± SE (N = 5-8).*; p < 0.05 HFD compared to LFD, $; p < 0.05 hyper compared to basal. HFD, high fat diet; LFD, low fat diet. |

## References

1. van den Berg, S.A., et al.*, High levels of dietary stearate promote adiposity and deteriorate hepatic insulin sensitivit*y. Nutr Metab (Lond), 2010**.** 7: p. 24.
