# Supplementary material for: High Fat Diet-Induced Changes in Mouse Muscle Mitochondrial Phospholipids Do Not Impair Mitochondrial Respiration Despite Insulin Resistance
Source: PLoS One. 2011 Nov 28;6(11):e27274. doi: 10.1371/journal.pone.0027274 (PMC3225362; doi:10.1371/journal.pone.0027274)
Supplement: Supporting Information S7 — IRS-1 serine phosphorylation in LFD (black bar) and HFD (white bar) mice after 8 weeks of dietary intervention. Briefly, equal amounts of muscle membrane protein fractions were loaded on SDS-PAGE. After Western blotting, membranes were incubated with an antibody detecting IRS-1 phosphorylation at Ser307 (#2381S, Cell Signaling Technology, Bioké, Leiden, The Netherlands). After incubation with the appropriate secondary IRDye680-labeled antibody (Licor, Westburg, Leusden, the Netherlands), the specific IRS-1 bands were detected and analyzed using the Odyssey near Infrared Scanner (Licor). Values are mean ± SE (n = 4). * p<0.05, assessed by independent samples t-test. The increased IRS-1 serine phosphorylation indicates the presence of muscle insulin resistance. (DOC) [file pone.0027274.s007.doc]

# Supporting Information 7

## High fat diet-induced changes in mouse muscle mitochondrial phospholipid composition and function are unrelated to insulin resistance

Joris Hoeks^1,*^, Janneke de Wilde^1,2*^, Martijn F.M. Hulshof^1,2^,Sjoerd .A.A. van den Berg^2,3^, Gert Schaart^4^, Ko Willems van Dijk^1,3,5^, Egbert Smit^1,2^, Edwin.C.M. Mariman^1,2^

* both authors contributed equally

^1^NUTRIM School for Nutrition, Toxicology and Metabolism, Department of Human Biology, Maastricht University Medical Center^+^, Maastricht, the Netherlands; ^2^Top Institute Food and Nutrition, Nutrigenomics Consortium, Wageningen, the Netherlands; ^3^Department of Human Genetics, University Medical Center Leiden, Leiden, the Netherlands; ^4^NUTRIM School for Nutrition, Toxicology and Metabolism, Department of Human Movement Sciences, Maastricht University Medical Center^+^, Maastricht, the Netherlands; ^5^Department of Internal Medicine, University Medical Center Leiden, Leiden, the Netherlands

| 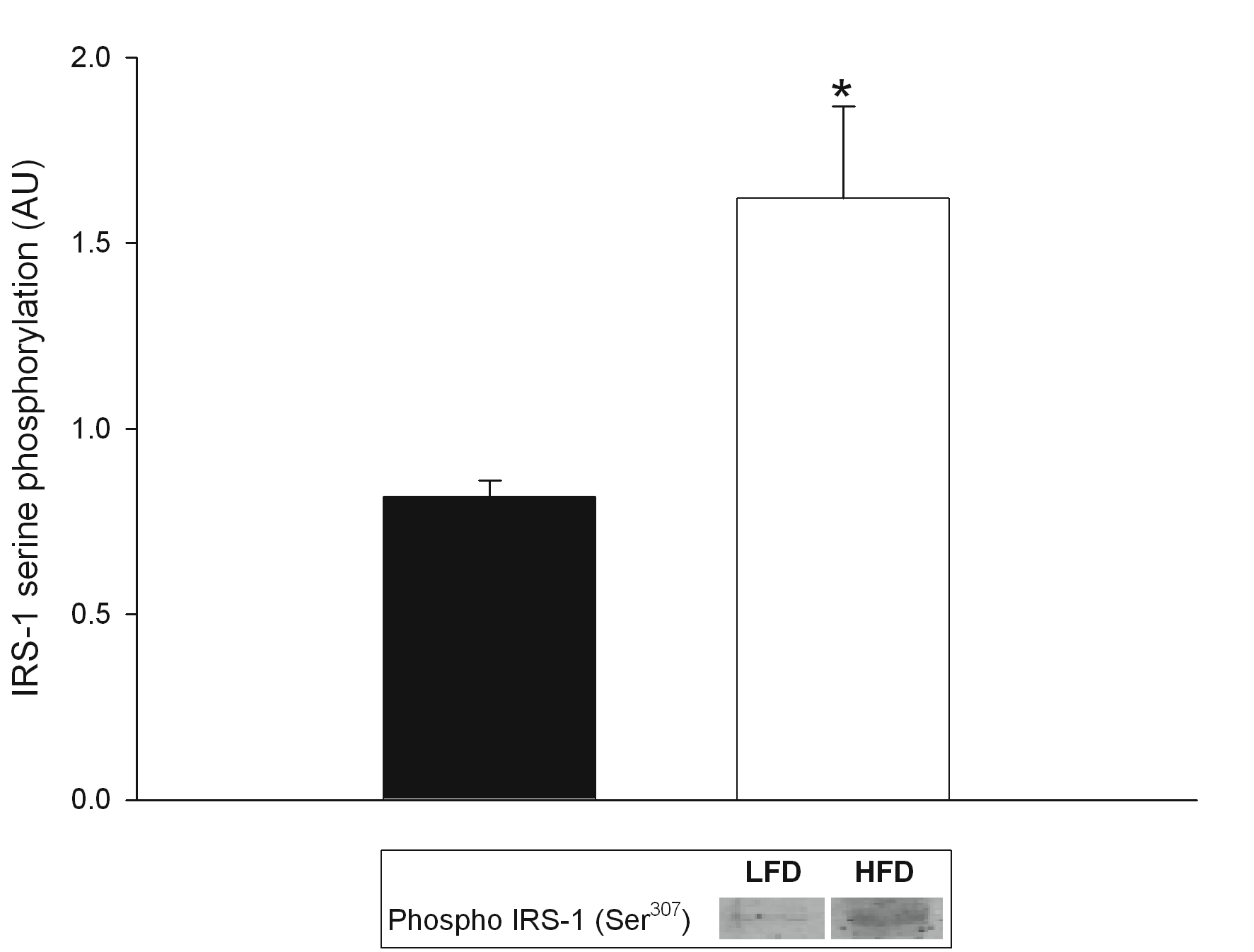 |
| --- |
| Supporting Information 7: IRS-1 serine phosphorylation in LFD (black bar) and HFD (white bar) mice after 8 weeks of dietary intervention.  Briefly, equal amounts of muscle membrane protein fractions were loaded on SDS-PAGE. After Western blotting, membranes were incubated with an antibody detecting IRS-1 phosphorylation at Ser^307^ (#2381S, Cell Signaling Technology, Bioké, Leiden, The Netherlands). After incubation with the appropriate secondary IRDye680-labeled antibody (Licor, Westburg, Leusden, the Netherlands), the specific IRS-1 bands were detected and analyzed using the Odyssey near Infrared Scanner (Licor). Values are mean ± SE (n=4). * p<0.05, assessed by independent samples t-test. The increased IRS-1 serine phosphorylation indicates the presence of muscle insulin resistance. |
