# Supplementary material for: High Fat Diet-Induced Changes in Mouse Muscle Mitochondrial Phospholipids Do Not Impair Mitochondrial Respiration Despite Insulin Resistance
Source: PLoS One. 2011 Nov 28;6(11):e27274. doi: 10.1371/journal.pone.0027274 (PMC3225362; doi:10.1371/journal.pone.0027274)
Supplement: Supporting Information S8 — Quantification of 4-HNE protein adducts in the quadriceps, gastrocnemius and TA muscle of mice fed an LFD or HFD for 8 and 20 weeks, respectively. Protein adducts of the lipid peroxidation byproduct 4-hydroxynonenal (4-HNE) were determined as marker of lipid peroxidation. Western blotting was performed as described [5]. We did not detect an increase in oxidative stress upon 8 or 20 weeks of HFD in any of the muscles studied. Thus, neither diet, nor time significantly changed the level of 4-HNE protein adducts in the quadriceps (A) as well as the TA muscle (C). In the gastrocnemius (B) we observed a significant diet * time effect with an increase in 4-HNE protein adducts over time in LFD mice, whereas a decrease over time was seen in HFD mice (LFD: 0.99 vs. 1.13 and HFD: 0.99 vs. 0.92 in 8-week vs. 20-week). Black bars and white bars represent LFD mice and HFD mice, respectively. Values (arbitrary units) are means ± SE (n = 6). D*T, significant diet * time effect with p<0.05; HFD, high fat diet; LFD, low fat diet; TA, tibialis anterior. (DOC) [file pone.0027274.s008.doc]

# Supporting Information 8

## High fat diet-induced changes in mouse muscle mitochondrial phospholipid composition and function are unrelated to insulin resistance

Joris Hoeks^1,*^, Janneke de Wilde^1,2*^, Martijn F.M. Hulshof^1,2^,Sjoerd .A.A. van den Berg^2,3^, Gert Schaart^4^, Ko Willems van Dijk^1,3,5^, Egbert Smit^1,2^, Edwin.C.M. Mariman^1,2^

* both authors contributed equally

^1^NUTRIM School for Nutrition, Toxicology and Metabolism, Department of Human Biology, Maastricht University Medical Center^+^, Maastricht, the Netherlands; ^2^Top Institute Food and Nutrition, Nutrigenomics Consortium, Wageningen, the Netherlands; ^3^Department of Human Genetics, University Medical Center Leiden, Leiden, the Netherlands; ^4^NUTRIM School for Nutrition, Toxicology and Metabolism, Department of Human Movement Sciences, Maastricht University Medical Center^+^, Maastricht, the Netherlands; ^5^Department of Internal Medicine, University Medical Center Leiden, Leiden, the Netherlands

| 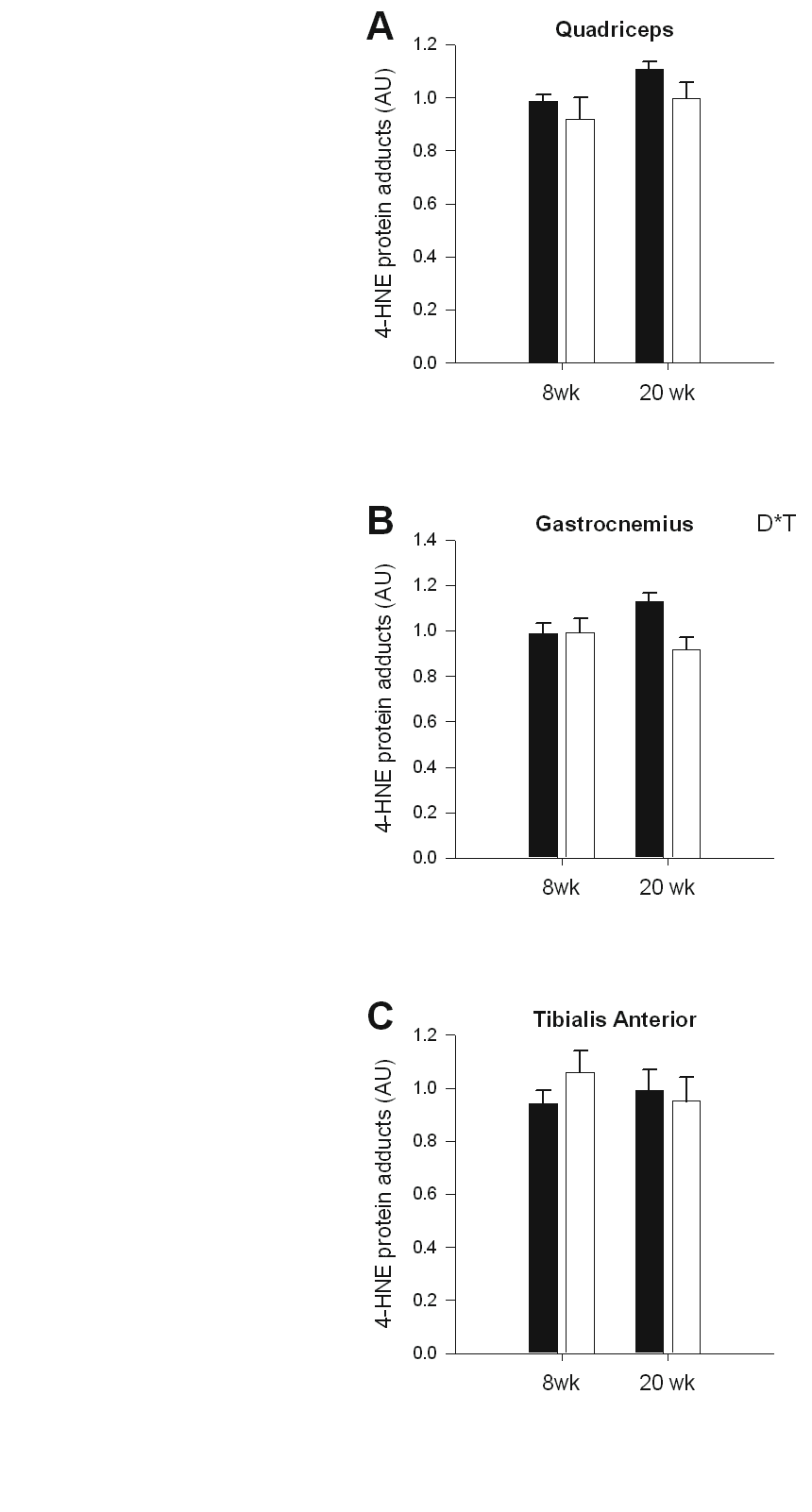 | 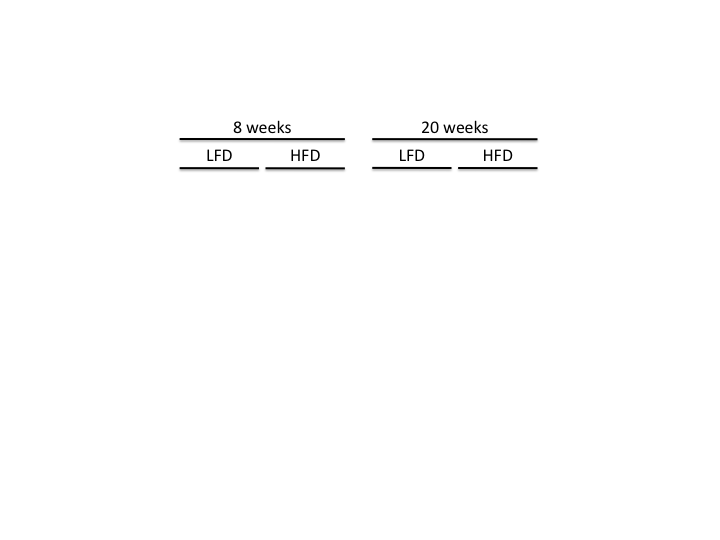 |
| --- | --- |
|  | 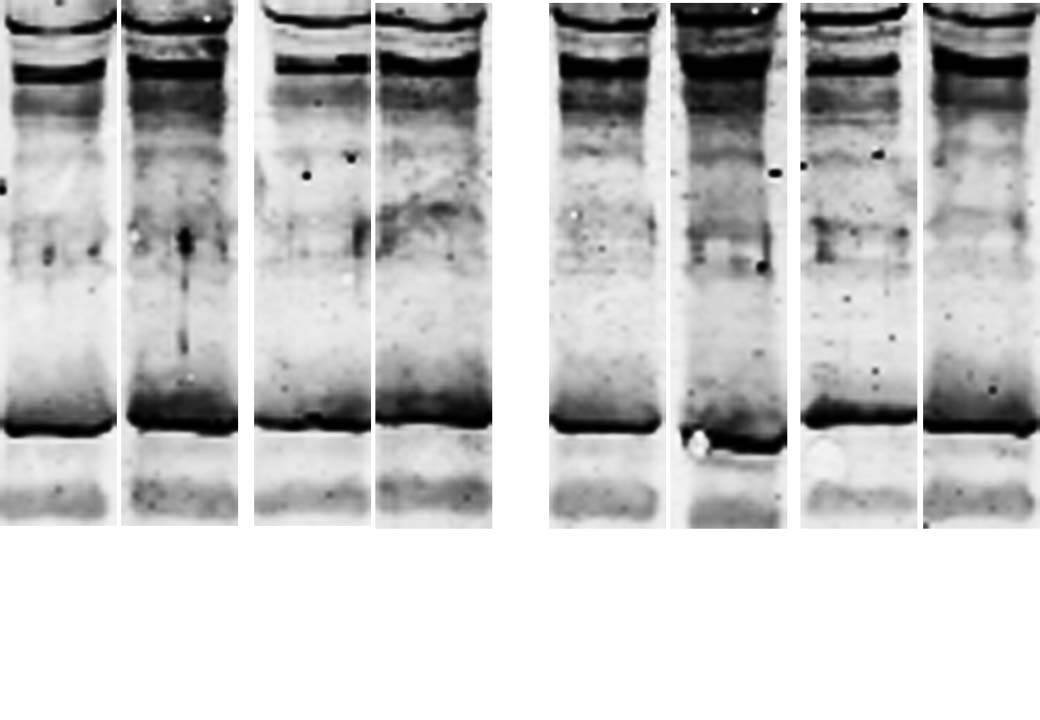 |
|  | 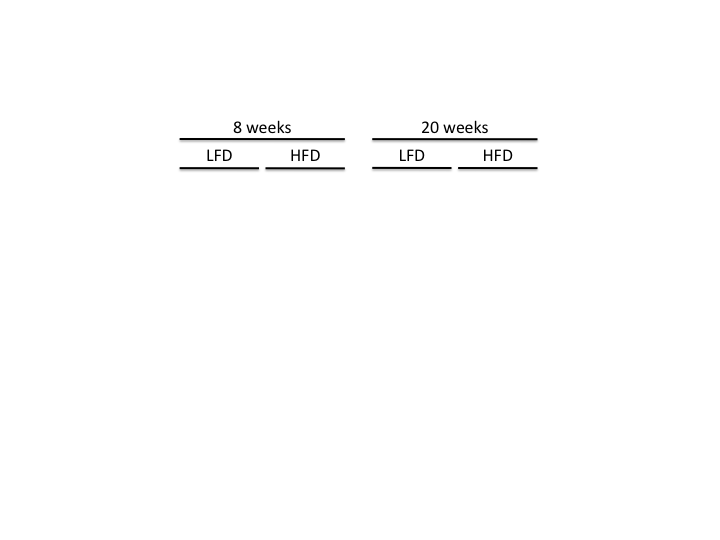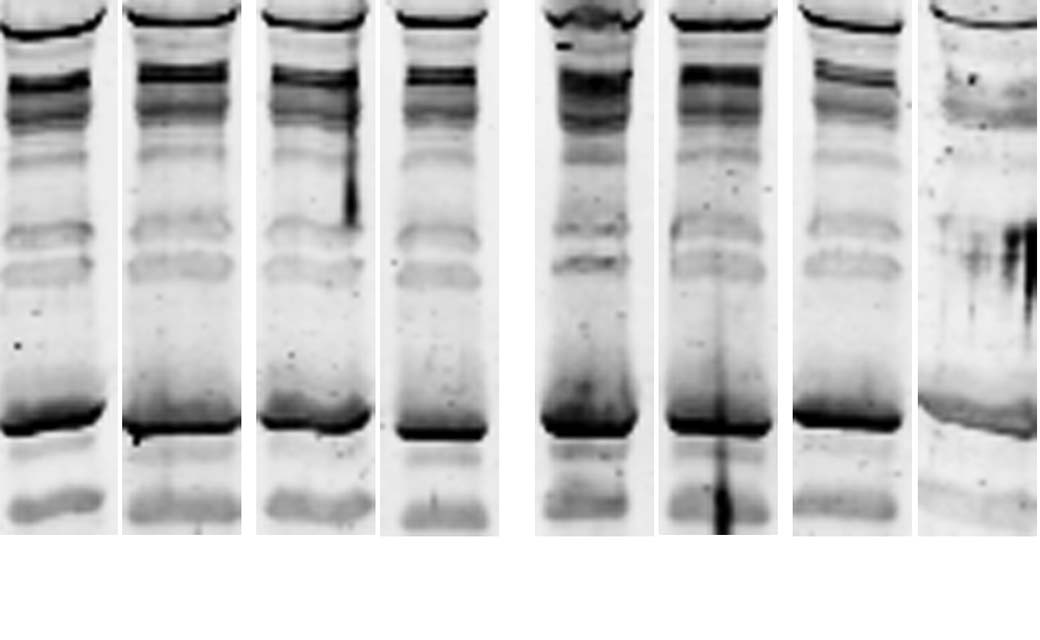 |
|  | 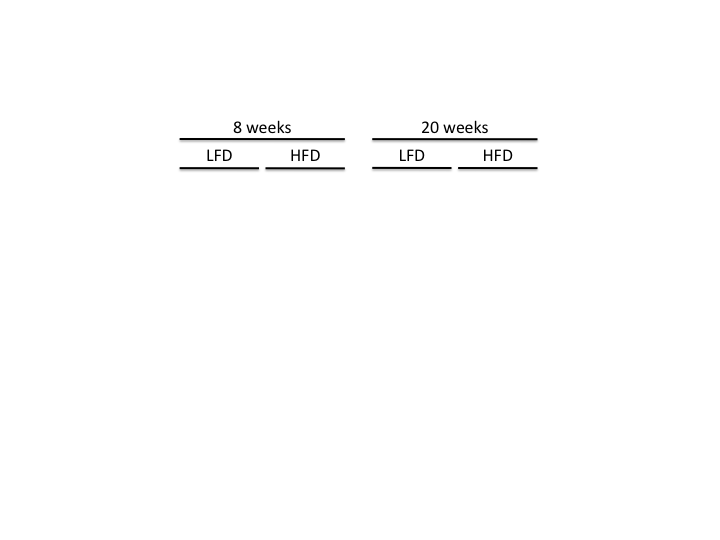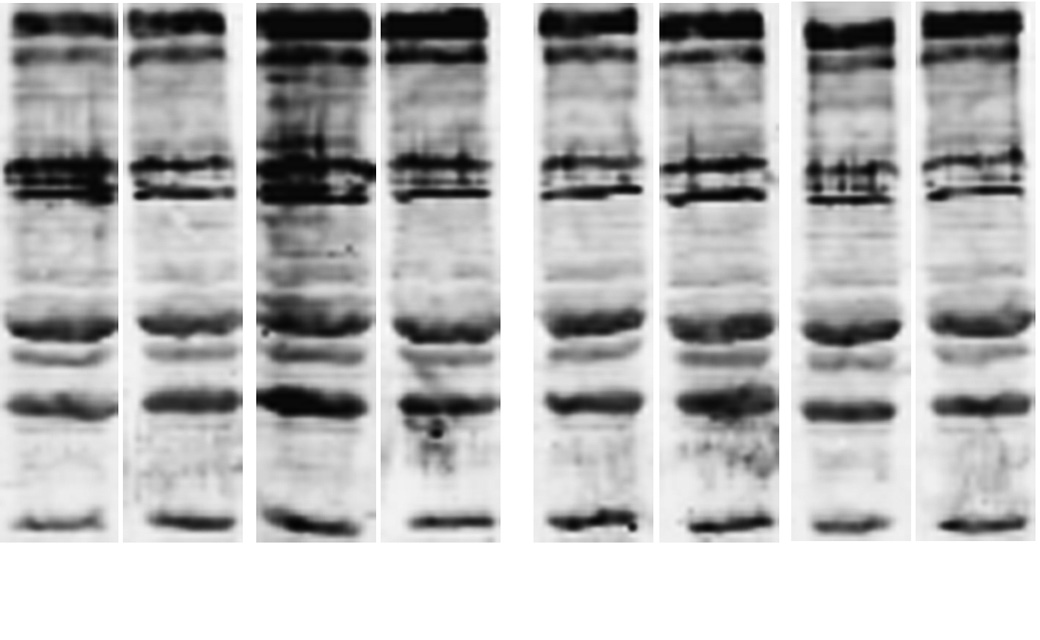 |
| Supporting Information 8: Quantification of 4-HNE protein adducts in the quadriceps, gastrocnemius and TA muscle of mice fed an LFD or HFD for 8 and 20 weeks, respectively.  Protein adducts of the lipid peroxidation byproduct 4-hydroxynonenal (4-HNE) were determined as marker of lipid peroxidation. Western blotting was performed as described [1]. We did not detect an increase in oxidative stress upon 8 or 20 weeks of HFD in any of the muscles studied. Thus, neither diet, nor time significantly changed the level of 4-HNE protein adducts in the quadriceps (A) as well as the TA muscle (C). In the gastrocnemius (B) we observed a significant diet * time effect with an increase in 4-HNE protein adducts over time in LFD mice, whereas a decrease over time was seen in HFD mice (LFD: 0.99 vs. 1.13 and HFD: 0.99 vs. 0.92 in 8-week vs. 20-week). Black bars and white bars represent LFD mice and HFD mice, respectively. Values (arbitrary units) are means ± SE (n = 6). D*T, significant diet * time effect with p < 0.05; HFD, high fat diet; LFD, low fat diet; TA, tibialis anterior. | |

## References

1. Nabben, M., et al.*, The effect of UCP3 overexpression on mitochondrial ROS production in skeletal muscle of young versus aged mic*e. FEBS Lett, 2008**. 5**82(30): p. 4147-52.
