# Supplementary material for: High Fat Diet-Induced Changes in Mouse Muscle Mitochondrial Phospholipids Do Not Impair Mitochondrial Respiration Despite Insulin Resistance
Source: PLoS One. 2011 Nov 28;6(11):e27274. doi: 10.1371/journal.pone.0027274 (PMC3225362; doi:10.1371/journal.pone.0027274)
Supplement: Supporting Information S9 — Fatty acid composition of palm oil. (DOC) [file pone.0027274.s009.doc]

# Supporting Information 9

## High fat diet-induced changes in mouse muscle mitochondrial phospholipid composition and function are unrelated to insulin resistance

Joris Hoeks^1,*^, Janneke de Wilde^1,2*^, Martijn F.M. Hulshof^1,2^,Sjoerd .A.A. van den Berg^2,3^, Gert Schaart^4^, Ko Willems van Dijk^1,3,5^, Egbert Smit^1,2^, Edwin.C.M. Mariman^1,2^

* both authors contributed equally

^1^NUTRIM School for Nutrition, Toxicology and Metabolism, Department of Human Biology, Maastricht University Medical Center^+^, Maastricht, the Netherlands; ^2^Top Institute Food and Nutrition, Nutrigenomics Consortium, Wageningen, the Netherlands; ^3^Department of Human Genetics, University Medical Center Leiden, Leiden, the Netherlands; ^4^NUTRIM School for Nutrition, Toxicology and Metabolism, Department of Human Movement Sciences, Maastricht University Medical Center^+^, Maastricht, the Netherlands; ^5^Department of Internal Medicine, University Medical Center Leiden, Leiden, the Netherlands

Supporting Information 9: Fatty acid composition of palm oil

| Fatty acid | (%) |
| --- | --- |
| 14:0 | 1 |
| 16:0 | 45 |
| 18:0 | 4 |
| 18:1 | 40 |
| 18:2 | 10 |

## 
